# The Effect of Adenotonsillectomy on Pediatric Nocturnal Enuresis: a Prospective Cohort Study

**Published:** 2013

**Authors:** Mohammad Saeed Ahmadi, Shahriar Amirhassani, Jalal Poorolajal

**Affiliations:** 1 *Department of Otolaryngology & Head and Neck surgery, School of Medicine, Hamadan University of Medical Sciences, Hamadan, Iran*.; 2 *Department of Urology, School of Medicine, Hamadan University of Medical Sciences, Hamadan, Iran.*; 3 *Research Center for Health Sciences , Department of Epidemiology & Biostatistics, School of Public Health, Hamadan University of Medical Sciences, Hamadan, Iran.*

**Keywords:** Adenoids, Cohort studies, Enuresis, Hypertrophy, Iran

## Abstract

**Introduction:**

Sleep disorder caused by adenotonsillar hypertrophy has been implicated as a cause of primary and secondary nocturnal enuresis in children. This study was conducted to investigate the effect of adenotonsillectomy on enuresis in children with adenotonsillar hypertrophy.

**Materials and Methods:**

This prospective cohort study was conducted in Hamadan City in Western Iran, from April 2010 to December 2011. Ninety-seven children aged 3 to 12 years with adenotonsillar hypertrophy who were admitted to Besat Hospital for adenotonsillectomy were evaluated. The primary outcome was the number of incidents of bedwetting (nocturnal enuresis) post-operation compared with pre-operation. Patients were followed-up for 3 months. Data were collected using a questionnaire regarding number of bedwetting incidents, type of enuresis (primary or secondary), and family history of enuresis, as well as results of urine analysis.

**Results:**

Of 420 children admitted for adenotonsillectomy, 97 had a positive history of preoperative enuresis, including 42 girls and 55 boys, with mean age of 48 months. The parents of 84 (86.6%) children agreed to participate in the study. Three months after adenotonsillectomy, enuresis had resolved completely in 51 (60.7%) children and had shown relative improvement in 22 (26.2%) children. Enuresis had not improved in the remaining 11 (13.1%) children (P<0.001).

**Conclusion:**

The results of this study indicate that adenotonsillectomy can improve enuresis in the majority of children with adenotonsillar hypertrophy. However, further evidence based on large multi-center randomized clinical trials is required to confirm these results.

## Introduction

Nocturnal enuresis (bedwetting) among children is one of the most common urological problems facing primary care providers today (1). The incidence rate of enuresis in 5-year-old children is estimated at approximately 15.1%, although underreporting makes the true incidence unknown. Fifteen percent of affected children will usually experience spontaneous resolution yearly, while 5% continue to experience nocturnal enuresis by the age of 10 years and 1% remain unimproved into adulthood (2). 

There is no definite and unambiguous etiology for nocturnal enuresis among children, and the disorder is probably multifactorial. Many potential causes have been suggested and investigated, such as dysfunction of sleep arousal, altered diurnal antidiuretic hormone secretion, genetic factors, nocturnal polyuria, psychological factors, delayed maturation, and parental age and education level (3,4). A correlation between sleep disorder and nocturnal enuresis among children has been proposed by previous case series and retrospective studies (5–7). Adenotonsillar hypertrophy is the most common cause of obstructive sleep apnea among children, and is an etiologic predictor for nocturnal enuresis. Several retrospective studies have addressed the beneficial effects of adenotonsillectomy in improving nocturnal incontinence in children with simultaneous adenotonsillar hypertrophy and preoperative enuresis (8–12). 

A recently published literature review regarding the beneficial effects of adenotonsillectomy indicated that upper airway obstruction and sleep disorder in children is frequently associated with nocturnal enuresis (13). This cohort study was performed to investigate prospectively the beneficial effect of adenotonsillectomy on nocturnal incontinence in children with adenotonsillar hypertrophy.

## Materials and Methods

This prospective cohort study was conducted from April 2010 to December 2012 in Hamadan City, West of Iran. The children, who were aged from 3 to 12 years with nocturnal enuresis as well as adenotonsillar hypertrophy with obstructive criteria, were admitted to Besat Hospital for adenotonsi- llectomy. For the purposes of this study, enuresis was defined as nighttime bedwetting or daytime incontinence to any degree in children older than 3 years and toilet trained. Children with urinary incontinence associated with a well-known urological or neurourological dysfunction and with primary enuresis were excluded from the study. Ninety-seven out of 420 children admitted for adenotonsillectomy were eligible. The parents of 84 (86.6%) children signed the informed consent form, including a child assent form for patients older than 7 years. Data collection toll was a questionnaire regarding the number of nighttime bedwetting and daytime incontinence episodes per week. Participants were followed-up and the same questionnaire was completed three months later. The Chi-square test was performed for data analysis at the 95% confidence level using statistical package Stata 11(StataCorp, College Station, TX, USA).

## Results

The mean age of the children included in this analysis was 48 months. The prevalence of nocturnal enuresis among children with adenotonsillar hypertrophy was 23.1% (97/420), with a prevalence of 25.2% (55/218) in boys and 20.8% (42/202) in girls (P=0.281) (Table 1).

**Fig 1:**
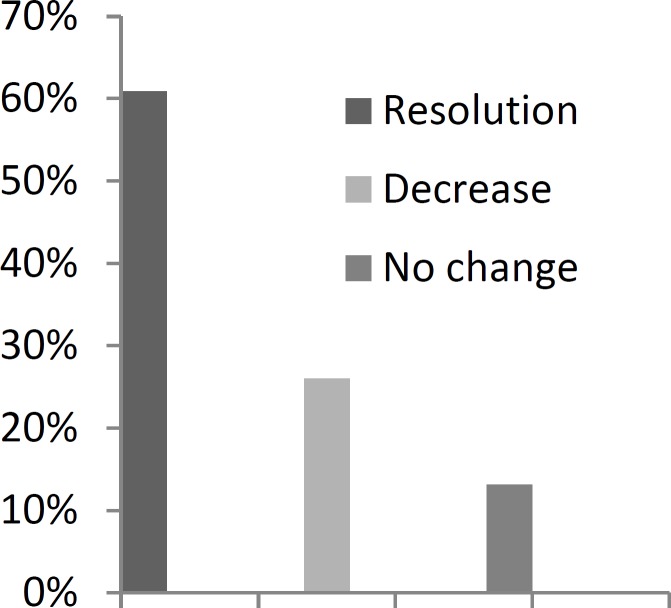
Effects of adenotonsillectomy on enuresis (N=84).

Of 84 children involved in the study, nocturnal enuresis and daytime incontinence was resolved completely in 51 (60.7%) children (P<0.001) (Fig 1). 

A relative improvement was observed in 22 (26.2%) children. The remaining 11 (13.1%) children showed no improvement after adenotonsillectomy.

**Table 1:** Baseline patient characteristics of the children admitted for adenotonsillectomy

**Gender**	**With enuresis** **Number (%)**	**Without ** **enuresis** **Number (%)**	**Total** **Number (%)**	**P value**
Male	55 (25.2)	163 (64.8)	218 (52)	0.281
Female	42 (20.8)	160 (79.2)	202 (48)	
Total	97 (23.1)	323 (76.9)	420 (100)	

## Discussion

The results of this study indicate that adenotonsillectomy can improve enuresis in the majority of children with adenotonsillar hypertrophy. Adenotonsillar hypertrophy is one the most common leading causes of upper airway obstruction which may result in nocturnal enuresis in children (13). The pathophysiological mechanism through which airway obstruction may cause enuresis is related to the increased work of breathing that can lead to higher negative intrathoracic pressure during inspiration and hence increased cardiac load. This effect may increase circulating serum levels of atrial and brain natriuretic peptides (14), which are produced and released by cardiac myocytes in response to stretch or strain, and result in diuresis (15).

Based on our findings, the prevalence of nocturnal enuresis in children with adenotonsillar hypertrophy was 23.1%. Brooks and Topol assessed 115 children with obstructive sleep apnea and reported a 41% prevalence of nocturnal enuresis (49% in boys and 31% in girls) (5). Basha S et al studied 326 children with obstructive sleep apnea (OSA) and reported that 32.8% of the children had various degrees of nocturnal enuresis (10). However, several anatomical, psychological, and hormonal factors as well as parental characteristics may play a role in the etiology of nocturnal enuresis (3,4).

Based on our results, the post-operation cure rate and improvement rate of enuresis symptoms were 60.7% and 26.2%, respectively. Basha S et al investigated the effect of adenotonsillectomy on nocturnal enuresis and found an 84.2% improvement in enuresis symptoms after surgery (10). Another study conducted by Weider et al in 115 children with nocturnal enuresis and OSA reported that enuresis symptoms were improved in 76% of patients after surgery (8). In a third study of 321 patients with enuresis who underwent adenotonsillectomy for upper airway obstruction, a cure rate of 63% and an improvement rate of 4% were reported after 3 months (9). Ahmet G et al studied 398 children with OSA secondary to adenotonsillar hypertrophy and reported complete resolution or partial improvement of enuresis in more than two-thirds of patients (16).

The greatest limitation of this study is that the parents of 13 out of 97 (13.4%) children refused to participate in the study. This issue may raise the possibility of selection bias in the study results.

## Conclusion

The results of this study indicate that adenotonsillectomy can improve enuresis in the majority of children with adenotonsillar hypertrophy. However, further evidence based on large multi-center randomized clinical trials is required to confirm these results.

## References

[1] Lawless MR, McElderry DH (2001). Nocturnal enuresis: current concepts. Pediatr Rev.

[2] Forsythe WI, Redmond A (1974). Enuresis and spontaneous cure rate. Study of 1129 enuresis. Arch Dis Child.

[3] Jalkut MW, Lerman SE, Churchill BM (2001). Enuresis. Pediatr Clin North Am.

[4] Akis N, Irgil E, Aytekin N (2002). Enuresis and the effective factors-a case-control study. Scand J Urol Nephrol.

[5] Brooks LJ, Topol HI (2003). Enuresis in children with sleep apnea. J Pediatr.

[6] Kaditis AG, Finder J, Alexopoulos EI, Starantzis K, Tanou K, Gampeta S (2004). Sleep-disordered breathing in 3,680 Greek children. Pediatr Pulmonol.

[7] Stone J, Malone PS, Atwill D, McGrigor V, Hill CM (2008). Symptoms of sleep-disordered breathing in children with nocturnal enuresis. J Pediatr Urol.

[8] Weider DJ, Sateia MJ, West RP (1991). Nocturnal enuresis in children with upper airway obstruction. Otolaryngol Head Neck Surg.

[9] Cinar U, Vural C, Cakir B, Topuz E, Karaman MI, Turgut S (2001). Nocturnal enuresis and upper airway obstruction. Int J Pediatr Otorhinolaryngol.

[10] Basha S, Bialowas C, Ende K, Szeremeta W (2005). Effectiveness of adenotonsillectomy in the resolution of nocturnal enuresis secondary to obstructive sleep apnea. Laryngoscope.

[11] Firoozi F, Batniji R, Aslan AR, Firoozi F, Batniji R, Aslan AR (2006). Resolution of diurnal incontinence and nocturnal enuresis after adenotonsillectomy in children. J Urol.

[12] Weissbach A, Leiberman A, Tarasiuk A, Goldbart A, Tal A (2006). Adenotonsilectomy improves enuresis in children with obstructive sleep apnea syndrome. Int J Pediatr Otorhinolaryngol.

[13] Leiberman A, Stiller-Timor L, Tarasiuk A, Tal A (2006). The effect of adenotonsillectomy on children suffering from obstructive sleep apnea syndrome (OSAS): the Negev perspective. Int J Pediatr Otorhinolaryngol.

[14] Kaditis AG, Alexopoulos EI, Hatzi F, Kostadima E, Kiaffas M, Zakynthinos E (2006). Overnight change in brain natriuretic peptide levels in children with sleep-disordered breathing. Chest.

[15] Potter LR, Yoder AR, Flora DR, Antos LK, Dickey DM (2009). Natriuretic peptides: their structures, receptors, physiologic functions and therapeutic applications. Handb Exp Pharmacol.

[16] Goke A, Aslan S, Yalcinkaya FR, Davarci M, Kaya YS, Savas N (2012). Improvement of monosymptomatic enuresis after adenoton- sillectomy in children with obstructive sleep apnea syndrome. Turk J Med Sci.

